# The Addition of Other Fecal Biomarkers Does Not Improve the Diagnostic Accuracy of Immunochemical Fecal Occult Blood Test Alone in a Colorrectal Cancer Screening Cohort

**DOI:** 10.3389/fmed.2021.665786

**Published:** 2021-06-04

**Authors:** Gonzalo Hijos-Mallada, Alberto Lué, Raul Velamazan, Nuria Saura, Carlos Abril, Marta Lorenzo, Mercedes Navarro, Eduardo Chueca, Samantha Arechavaleta, Fernando Gomollón, Ángel Lanas, Carlos Sostres

**Affiliations:** ^1^Digestive Diseases Service, University Clinic Hospital, Zaragoza, Spain; ^2^Aragón Health Research Institute (IIS Aragón), Zaragoza, Spain; ^3^University of Zaragoza, Zaragoza, Spain; ^4^CIBER Enfermedades Hepáticas y Digestivas (CIBERehd), Zaragoza, Spain

**Keywords:** colorrectal cancer, screening, fecal occult blood, fecal calprotectin, fecal transferrin, fecal lactoferrin, rapid tests

## Abstract

**Background:** Screening with fecal occult blood test reduces colorectal cancer (CRC) incidence and mortality, and is currently implemented in most countries. However, around 40% of screening colonoscopies are normal. Thus, strategies to avoid these colonoscopies are highly necessary. Adding other fecal biomarkers, such as fecal calprotectin (FC), lactoferrin, and transferrin may be useful, but evidence is scarce.

**Aims:** To evaluate the diagnostic accuracy of fecal occult blood immunochemical test (FIT), FC, and a one-step combo card test for the simultaneous semi-qualitative detection of human hemoglobin (hHb), transferrin (hTf), calprotectin (hCp) and lactoferrin (hLf) in a CRC screening program population.

**Methods:** Single-center, prospective observational study, enrolling patients included in a CRC screening program, referred for a colonoscopy due to a positive FIT test. Participants collected a stool sample prior to bowel preparation, and FIT, FC and the combo semi-qualitative tests were performed on the sample. Sensitivity, specificity, positive and negative predictive values and area under receiver operator curve (AUC) for diagnosis of advanced neoplasia, advanced adenoma and CRC were estimated for each biomarker and their combinations. The primary endpoint of the study was to assess whether these biomarkers could improve the diagnostic accuracy of FIT alone.

**Results:** 336 consecutive patients (64% males) were recruited. Advanced neoplasia was found in 129/336 (38.4%) patients, and of these, 22/336 (6.5%) were diagnosed of CRC. 153/336 (45.5%) colonoscopies were completely normal. The AUC for the diagnosis of advanced neoplasia were 0.725 (95%CI 0.665–0.784) for FIT, 0.477 (95%CI 0.413–0.541) for FC and 0.732 (95%CI 0.674–0.791) for the combination of both (FIT + FC) quantitative tests. The AUCs for the combo test were 0.70 (95%CI 0.641–0.760) for hHb, 0.625 (95%CI 0.562–0.698) for hTf, 0.532 (95%CI 0.469–0.595) for hCp, 0.531 (95%CI 0.466–0.595 ) for hLf and 0.681 (95%CI 0.620–0.741) for the combination of the four biomarkers.

**Conclusion:** In average-risk population, FIT appears to be the best fecal marker for the diagnosis of CRC and advanced adenoma. None of the other biomarkers explored or their combinations provided a better diagnostic accuracy. Only hTF showed an acceptable diagnostic accuracy. FC and hLF were not useful in this setting.

## Introduction

Colorectal cancer (CRC) is the third most commonly diagnosed cancer and the second in terms of mortality worldwide (1). Its incidence is expected to increase globally in the following years (2). Male sex and advanced age are well-recognized risk factors for CRC, as well as family history and environmental factors (3). CRC screening in averaged risk population using fecal occult blood test, followed by colonoscopy if positive, reduces both its incidence and mortality, being a cost-effective strategy (4–6). The fecal immunochemical test (FIT) it is now recommended as the first option for the CRC screening programs due to its clear advantages over the guaiac based test (7, 8). Its cut-off value can be chosen according the availability of endoscopic resources and the epidemiology of CRC in each population (9, 10). Based on this evidence, most European countries with CRC screening programs are currently using FIT with cut-off values between 20 and 30 μgr/gr (11).

However, FIT is not a perfect test (12). CRC screening programs around Europe have reported a 9.8–33.5% of advanced adenomas and 2.16–10.1% of CRC in colonoscopies performed (11), which means that a considerable proportion of these colonoscopies did not find any relevant pathology. Consequently, these patients without pathology are exposed to the non-negligible risk of endoscopy-related complications (13), and colonoscopy waiting lists are considerably increased (14). In order to improve the diagnostic accuracy of FIT in CRC screening, several strategies have been proposed.

The options of increasing the FIT cut-off (15, 16) or repeating FIT (17, 18) have been already explored. However, data regarding diagnostic accuracy of other fecal biomarkers in this setting are scarce. Fecal calprotectin (FC) is a biomarker which correlates well with bowel inflammation. It is widely used for diagnosis and monitoring of inflammatory bowel disease (19, 20). Data regarding its diagnostic accuracy in symptomatic population are variable (21–23). However, there is almost no evidence regarding its diagnostic accuracy in an average risk screening population. In 2004, FC test was compared with FIT in a CRC screening program cohort, concluding that FC cannot be recommended in this setting due to an insufficient adenoma detection rate (24). Fecal transferrin is released together with hemoglobin when bleeding and it has been suggested to increase the sensitivity of FIT. However, in a study published in 2018, in a CRC screening cohort neither transferrin nor its combination with FIT had a better diagnostic accuracy than FIT alone (25). Fecal lactoferrin, similar to FC, has mainly been used to evaluate activity in inflammatory bowel disease patients (26). In a study performed in 872 patients referred for colonoscopy, lactoferrin showed a sensitivity of 50% for CRC and 15.9% for advanced adenoma (27).

In this study, we analyze these four fecal biomarkers, alone and in combination, in a CRC screening population referred for colonoscopy (due to a previous positive FIT), in order to explore whether they can enhance the diagnostic accuracy of FIT alone.

## Materials and Methods

### Study Population

We performed a single-center, prospective observational study, enrolling patients included in the CRC screening program of the health area of University Clinic Hospital Lozano Blesa (Zaragoza), a general tertiary hospital. CRC screening program in our area is performed with FIT (FOB Gold®, SENTiFIT, Sysmex-Sentinel CH. SpA, Barcelona, Spain), a different test than the one evaluated in this study, with a cut-off of 20 μgr/gr. CRC screening program has been started in the 60 to 70 aged population, so all patients included belong to this age group Colonoscopy is indicated to all positive FIT patients.

CRC screening participants referred for colonoscopy because of a previously positive FIT result between January and June 2018 were consecutively enrolled in the study. Patients were contacted by the investigators approximately 1 week before colonoscopy was scheduled to inform about the study. Those who agreed to participate, were asked to collect a stool sample the day before starting colonic preparation, keep it refrigerated and bring it to the hospital the day of the colonoscopy. Every patient signed a written informed consent before being included in the study. Ethical approval was granted by the local ethic committee (CEICA–Regional Ethical Committee of Aragón).

Patients were excluded for the final analysis if the colonoscopy was requested for other indication (FIT in symptomatic patients, adenoma or CRC follow-up, family or personal history of CRC, polyposis, and inflammatory bowel disease follow-up), if the stool sample returned was insufficient or unsuitable for the analysis or if they had not signed the informed consent.

### Fecal Tests

The following fecal tests were performed:

1. FIT, by FOB Turbilatex® (Certest Biotec S.L, Zaragoza, Spain). Several cut-offs (5 μgr/gr, 20 μgr/gr and any detectable hemoglobin) were used for this study.
2. FC, by Calprotectin Turbilatex® (Certest Biotec S.L, Zaragoza, Spain), with a cut-off of 50 μgr/gr.
3. FOB+Transferrin+Calprotectin+Lactoferrin® (Certest Biotec S.L, Zaragoza, Spain), a one-step colored chromatographic inmmunoasay for the simultaneous semi-qualitative detection of human hemoglobin (hHb), human transferrin (hTf), human calprotectin (hCp) and human lactoferrin (hLf). Cut-off values of the test were 5.1 μg/gr for hHb, 0.4 μg/gr for hTf, 50 μg/gr for hCp and 10 μg/gr for hLf. Results can be only “positive” or “negative.” The test was performed and read by trained investigators.

### Colonoscopy and Definitions

We defined advanced neoplasia as the presence of either CRC or advanced adenoma (any adenoma ≥10 mm, with villous component or high grade dysplasia, or ≥ 3 adenomas) (28). Non-advanced adenomas as well as other non-neoplastic pathology were also registered and included in the final analysis. All diagnoses were confirmed histologically.

### Endpoint of the Study

The primary endpoint was to assess whether any of these biomarkers or their available combinations could improve the diagnostic accuracy of FIT alone for the diagnosis of advanced neoplasia in an average-risk population (individuals aged 50 years or older without other risk factors).

Secondary endpoints were to describe the diagnostic accuracy of these biomarkers individually for diagnosis of advanced adenoma and CRC, and to evaluate the prevalence of advanced adenoma and CRC in our CRC screening population.

### Statistical Analysis

A descriptive analysis of the patients included was performed. Continuous variables were expressed as mean with standard deviation or median with interquartile range. Qualitative variables were described with frequencies and percentages. Kolmogorov-Smirnov test was used to assess if continuous variables followed a normal distribution. Chi-square, Kruskal-Wallis and Mann-Whitney U tests were used to evaluate the relationship between different variables. Sensitivity, specificity, positive predictive value (PPV), negative predictive value (NPV) and area under receiver operator curve (AUC) were calculated for each fecal test and for their possible combinations, to detect advanced neoplasia, CRC and advanced adenoma. A logistic regression analysis was performed to calculate the AUC of FIT combined with FC (both quantitative variables). The method of DeLong et al. (29) was used to test the statistical significance of the differences between AUCs. SPSS version 26 and MedCalc version 13.3 were used for statistical analysis.

We calculated the sample size for this study based on the results of a pilot study carried out in 173 patients, which obtained a 33.5% prevalence of advanced neoplasia. Sensitivity and specificity results were 81 and 54.8% for FIT; 56.9 and 40% for FC. A sample size of 273 patients would be necessary to estimate the diagnostic accuracy of these tests and find differences between them, with a confidence level of 95% and a power of 80%. EPIDAT version 3.1 was used to calculated sample size.

## Results

### Baseline Characteristics of Patients

A total of 365 patients were contacted, of whom 341 agreed to participate in the study (93.4% participation rate). Five patients were excluded due to exclusion criteria (colonoscopy requested due to gastrointestinal symptoms or family history of CRC). Thus, 336 participants were included in the final analysis. Median age was 64 years (interquartile range 61–67 years), and 215 (64%) were male. Regarding concomitant treatments, 33 (9.8%) were active non-steroidal anti-inflammatory drugs users, 46 (13.7%) were taking low dose aspirin, 8 (2.4%) other antiplatelets, 11 (3.3%) were anticoagulated with acenocumarol and 12 (3.6%) with new oral anticoagulants.

### Colonoscopy Findings and Fecal Tests

Advanced neoplasia was detected in 129 (38.4%) of the participants. Of these, 107 (31.8%) were diagnosed with advanced adenomas and 22 (6.5%) with CRC. Non-advanced adenomas were found in 54 (16.1%) patients. A total of 153 (45.5%) colonoscopies were completely normal.

Positivity rates of the quantitative fecal tests were 81.8, 43.2, and 21.7% for FIT using 0 μg/gr, 5 μg/gr and 20 μg/gr as cut-offs, respectively, and 55.7% for FC. Regarding the 4 biomarkers combo test, positivity rates were 38.1% for hHb, 28% for hTf, 64.3% for hCp, 8.6% for hLf and 84.5% when any of them was positive.

### Diagnostic Accuracy of Fecal Tests

The sensitivity, specificity and predictive values of FIT with different cut-offs, FC and their possible combinations for detection of advanced neoplasia, CRC and advanced adenoma are summarized in Table 1. Median values of FIT and FC results in CRC, advanced adenoma, non-advanced adenoma and normal colonoscopies are represented in Table 2. For the four simultaneous biomarker semi-qualitative combo test, the sensitivity, specificity and predictive values of each biomarker and their combination are summarized in Table 3. When analyzing the combination of different tests, we have considered as a positive result if any of the tests turned positive.

**Table 1:** Diagnostic accuracy of quantitative FIT, FC and its combination.

		**FIT**	**FIT**	**FIT**	**FIT**	**FIT**	**FIT**	**FC**
		**> 0 μg/gr**	**>0 μg/gr + FC**	**> 5 μg/gr**	**> 5 μg/gr + FC**	**> 20 μg/gr**	**> 20 μg/gr + FC**	
**Number of positive tests**		***n* = 275**	***n* = 301**	***n* = 145**	***n* = 241**	***n* = 73**	***n* = 219**	***n* = 187**
Advanced neoplasia *n* = 129 (38.4%)	Sensitivity	86.82%	92.24%	64.34%	81.39%	42.63%	68.99%	50.38%
	Specificity	21.25%	12.07%	70.04%	34.29%	91.30%	37.68%	41.06%
	PPV	40.72%	39.53%	57.24%	43.56%	75.34%	40.82%	34.75%
	NPV	72.13%	71.42%	75.91%	74.74%	71.86%	66.1%	57.04%
	*p*-value	p = 0.062	*p* = 0.207	*p* < 0.001	*p* = 0.02	*p* < 0.001	*p* = 0.247	*p* = 0.125
	OR (95% CI)	1.78 (0.97–3.27)	1.63 (0.75–3.52)	4.22 (2.65–6.73)	2.28 (1.34–3.87)	7.84 (4.3–14.1)	1.32 (0.83–2.1)	0.71 (0.45–1.1)
CRC *n* = 22 (6.5%)	Sensitivity	95.45%	100%	86.36%	100%	72.72%	90.91%	63.63%
	Specificity	19.10%	11.14%	59.87%	30.25%	81.84%	36.94%	44.90%
	PPV	7.6%	7.3%	13.1%	9.13%	21.91%	9.17%	7.48%
	NPV	98.36%	100%	98.42%	100%	97.72%	98.31%	94.63%
Advanced adenoma *n* = 107 (31.8%)	Sensitivity	85.04%	90.65%	59.81%	77.57%	36.44%	64.48%	47.66%
	Specificity	19.65%	10.92%	64.62%	31%	85.15%	34.36%	40.61%
	PPV	33.09%	32.22%	44.13%	34.44%	53.42%	31.65%	27.27%
	NPV	73.77%	71.43%	77.48%	74.73%	74.14%	67.24%	62.41%

*FIT, fecal immunochemichal test; FC, Fecal calprotectin; PPV, positive predictive value; NPV, negative predictive value; CRC, colorectal cancer; OR, odds ratio, risk of presenting pathology with a positive fecal test compared to a negative result; CI, confidence interval. “p-values” were calculated using χ2 test*.

**Table 2:** Median values of FIT and FC results in advanced neoplasia, CRC, advanced adenomas, non-advanced adenomas and normal colonoscopies.

	**FIT. Median (IQR)**	***p-*value**	**FC. Median (IQR)**	***p-*value**
Advanced neoplasia	12.7 (68.19) μg/gr	*p* < 0.001	51.59 (108.4) μg/gr	*p* = 0.472
No advanced neoplasia	2.24 (5.3) μg/gr		61.54 (107.4) μg/gr	
CRC	112.75 (420.86) μg/gr	*p* < 0.001	69.79 (125) μg/gr	*p* = 0.316
Advanced adenoma	9.10 (37.40) μg/gr	*p* = 0.05	47 (104.3) μg/gr	
Non-advanced adenoma	4 (7.70) μg/gr	*p* = 0.09	61.01 (84.2) μg/gr	
Normal colonoscopies	2.08 (4.9) μg/gr	*p* < 0.001 vs. AA.	61.54 (109) μg/gr	

*FIT, fecal immunochemichal test; FC, Fecal calprotectin; IQR, interquartile range; AA, advanced adenoma; CRC, colorectal cancer. Kolmogorov-Smirnov test was used to conclude that neither FIT nor FC values follow a normal distribution. “p-values” were calculated using Kruskal-Wallis and Mann-Whitney U tests. Mann-Whitney U test was performed to evaluate the differences of FIT/FC values between the two main findings (advanced neoplasia vs. no advanced neoplasia). Kruskal-Wallis test was used to compare the FIT/FC values among the four possible diagnoses. As the “p-value” for FIT was <0.001 (not shown in the table), Mann-Whitney U test was performed to evaluate the differences of FIT concentration among each possible diagnosis pairwise*.

**Table 3:** Diagnostic accuracy of the four biomarker semi-qualitative combo test.

		**hHb**	**hTf**	**hCp**	**hLf**	**hHb + hCp**	**hHb + hTf**	**zero tests positive**	**four tests positive**
**Number of positive tests**		***n* = 128**	***n* = 94**	***n* = 216**	***n* = 29**	***n* = 249**	***n* = 153**	***n* = 256**	***n* = 15**
Advanced neoplasia *n* = 129 (38.4%)	Sensitivity	62.79%	43.41%	68.22%	12.4%	82.94%	65.89%	84.49%	10.07%
	Specificity	77.29%	81.64%	38.16%	93.71%	31.40%	67.15%	28.98%	99.03%
	PPV	63.28%	59.57%	40.74%	55.17%	42.97%	55.55%	42.58%	86.67%
	NPV	76.92%	69.83%	65.83%	63.19%	74.71%	75.96%	75%	63.86%
	*p*-value	*p* < 0.001	*p* < 0.001	*p* = 0.235	*p* = 0.052	*p* = 0.04	*p* < 0.001	*p* = 0.05	*p* < 0.001
	OR	5.74	3.41	1.32	2.11	2.22	3.95	0.45	11.49
	(95% CI)	(3,54–9.31)	(2.1–5.6)	(0.81–2.11)	(0.98–4.55)	(1.29–3.84)	(2.48–6.29)	(0.25–0.79)	(2.55–51.79)
CRC *n* = 22 (6.5%)	Sensitivity	90.91%	77.27%	95.45%	22.73%	100%	90.91%	100%	18.18%
	Specificity	65.60%	75.47%	37.9%	92.35%	27.71%	57.64%	25.47%	96.57%
	PPV	15.62%	18.08%	9.72%	17.24%	8.83%	13.07%	8.59%	26.67%
	NPV	99.04%	97.93%	99.16%	94.46%	100%	98.91%	100%	94.51%
Advanced adenoma *n* = 107 (31.8%)	Sensitivity	57%	36.45%	62.62%	10.28%	79.43%	60.75%	81.3%	8.41%
	Specificity	70.74%	75.98%	34.93%	92.14%	28.38%	61.57%	26.2%	97.38%
	PPV	47.65%	41.49%	31.02%	37.93%	34.14%	42.48%	33.98%	60%
	NPV	77.88%	71.91%	66.67%	68.73%	74.71%	77.05%	75%	69.47%

*hHb, human hemoglobin; hTf, human transferrin; hCp, human calprotectin; hLf, human lactoferrin; CRC, colorectal cancer; PPV, positive predictive value; NPV, negative predictive value; CI, confidence interval; Zero tests positive, negative results in the four biomarkers of the combo test (hHb, hTf, hCp, hLf); four tests positive, positive results in the four biomarkers of the combo test (hHb, hTf, hCp, hLf); OR, odds ratio, risk of presenting pathology with a positive fecal test compared to a negative result. “p-values” were calculated using χ2 test*.

### Receiver Operator Curve Analysis

**Figure 1:**
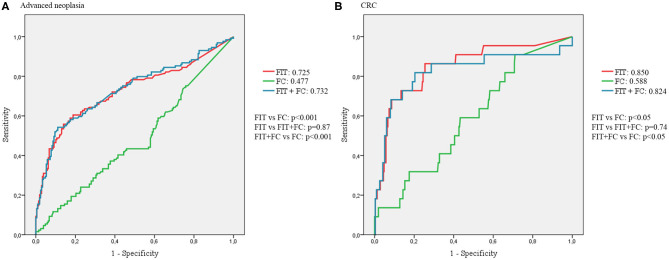
AUCs of FIT, FC and its combination. **(A)** AUCs for the diagnosis of advanced neoplasia. **(B)** AUCs for the diagnosis of CRC.

The AUC for advanced neoplasia detection were 0.725 (95%CI 0.665–0.784) for FIT, 0.477 (95%CI 0.413–0.541) for FC and 0.732 (95%CI 0.674–0.791) for the combination of both quantitative tests. For CRC diagnosis, the values were 0.850 (95%CI 0.758–0.943) for FIT, 0.588 (95%CI 0.471–0.705) for FC, and 0.824 (95%CI 0.706–0.942) for their combination. AUC of FIT, FC and their combination are represented in Figure 1.

**Figure 2:**
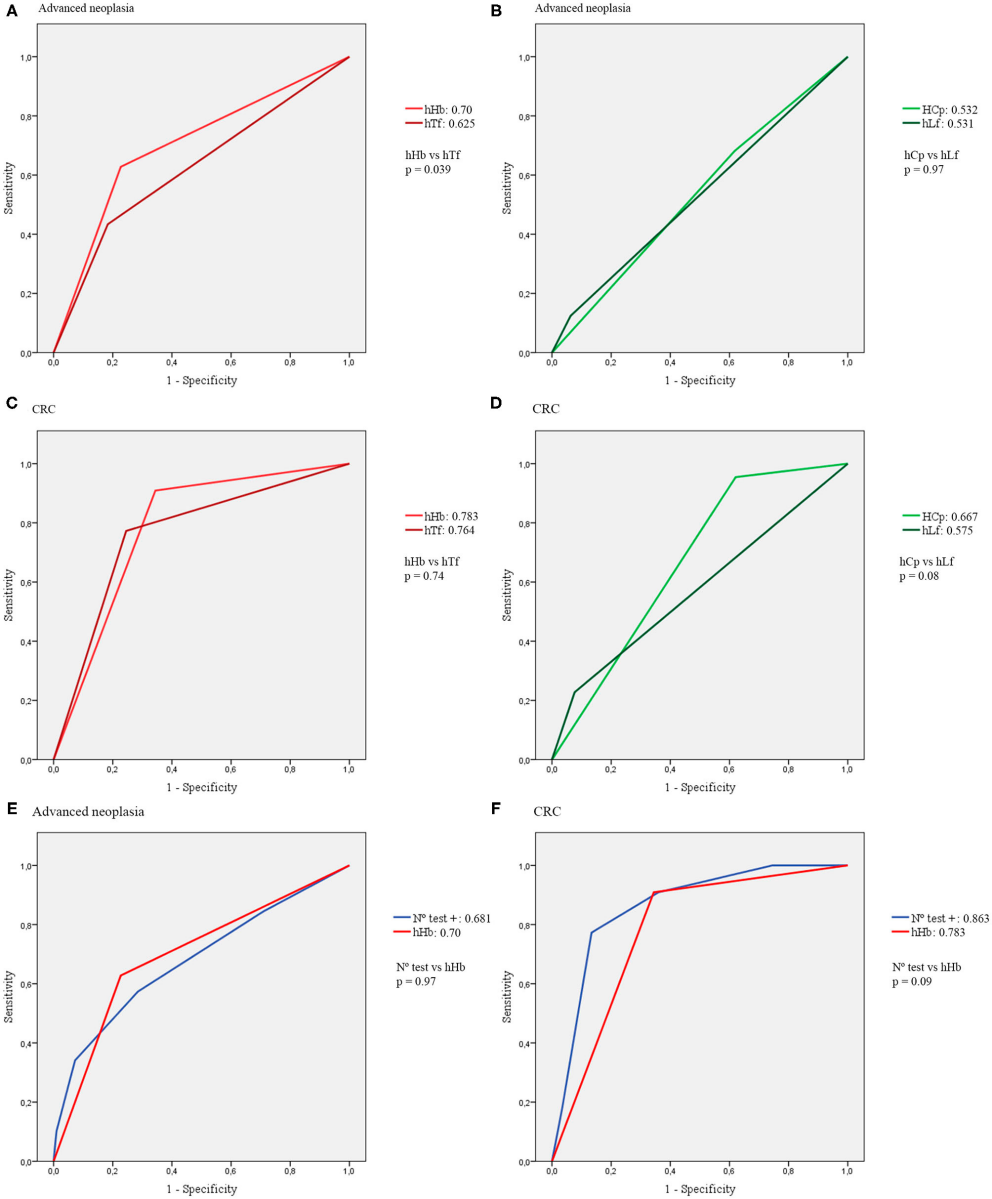
AUCs of the four biomarkers of the combo test. **(A)** AUCs of hHb and hTf for advanced neoplasia. **(B)** AUCs of hCp and hLf for advanced neoplasia. **(C)** AUCs of hHb and hTf for CRC. **(D)** AUCs of hCp and hLf for CRC. **(E)** AUCs of the combo test compared with hHb for advanced neoplasia. **(F)** AUC of the combo test compared with hHb for CRC.

Regarding the four biomarker simultaneous semi-qualitative combo test, the AUCs are summarized in Table 4 and represented in Figure 2. The AUC for the combination of the four tests was calculated considering the four possible cut-offs according to the number of positive tests.

**Table 4:** AUCs (95%CI) of the four biomarkers of the combo test and their combination for diagnosis of advanced neoplasia and CRC.

	**hHb**	**hTf**	**hCp**	**hLf**	**Combination of four test**
Advanced neoplasia	0.70 (0.641–0.760)	0.625 (0.562–0.698)	0.532 (0.469–0.595)	0.531 (0.466–0.595)	0.681 (0.620–0.741)
CRC	0.783 (0.7–0.865)	0.764 (0.659–0.869)	0.667 (0.574–0.76)	0.575 (0.441–0.710)	0.863 (0.794–0.932)

*AUC, Area under receiver operator curve; CI, confidence interval; hHb, human hemoglobin; hTf, human transferrin; hCp, human calprotectin; hLf, human lactoferrin; CRC, colorectal cancer*.

## Discussion

In this study, we have evaluated the diagnostic accuracy of 6 different fecal tests, in a CRC screening program population, with a previous positive FIT test. We have found a CRC prevalence of 6.5%. A figure that should be highlighted is that 45.5% of the colonoscopies performed were normal. Similar percentage has been reported in other CRC screening programs worldwide (11), a fact that points out the need for new strategies to improve the detection of patients with higher risk of CRC and adenoma and to avoid performing these normal colonoscopies.

In our cohort, quantitative FIT was the test with the best diagnostic accuracy, with an AUC of 0.725 for advanced neoplasia and 0.850 for CRC, both significantly higher that the AUC of all the other tests, and similar to the figures obtained in other studies in analogous population (25). Significant differences in the fecal hemoglobin concentration were found between patients with and without advanced neoplasia, and also between patients with CRC and advanced adenoma, confirming the fact that a higher hemoglobin concentration correlates with the risk of relevant pathology (9, 10). However, one of the 22 cases of CRC had undetectable FIT and three cases had FIT <5 μg/gr. All these cases (as every patient enrolled in this study) had had a previous positive FIT. This discrepancy of FIT has been reported before, being as high as 25% in patients with CRC and 42% with advanced adenoma (17). Nevertheless, we should remark that a high percentage of our cohort had a negative FIT result (78.3% < 20 μg/gr, 56.8% < 5 μg/gr and 18.2% undetectable). This is an unexpected result as all patients included had a previous positive FIT with a cut-off point of 20 μg/gr. This finding can lead to further investigation in this setting.

In this CRC screening population, FC did not prove to be a useful diagnostic tool, with an AUC < 0.5 and no significant differences found in FC levels between patients with or without advanced neoplasia. Same conclusion was reported by Hoff et al. in a study performed in a similar population (24). Therefore, adding FC to FIT did not improve the diagnostic accuracy of FIT. Adding FC to FIT raised slightly the sensitivity of FIT alone, as the highest value of sensitivity of all the possible combinations was reached combining any detectable FIT and FC, but this was paralleled by a low specificity, not improving the NPV of FIT alone. Therefore, adding FC to FIT does not seem to be an adequate strategy to avoid unnecessary colonoscopies in a CRC screening setting. However, it should be taken into consideration that the combination of FIT (with either any detectable value or 5 μg/gr as cut-offs) with FC was the only strategy detecting all the 22 CRC cases in this cohort (NPV 100%) using the quantitative fecal tests.

**Figure 3:**
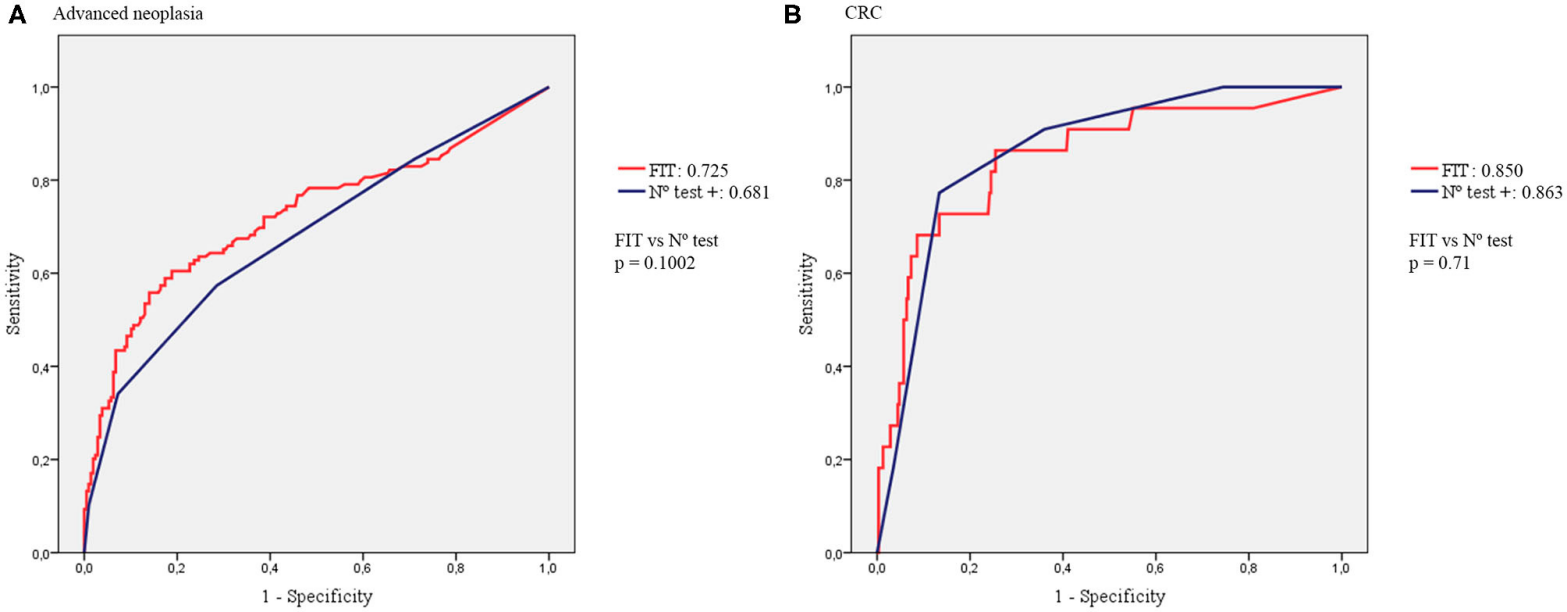
AUCs of FIT compared with the combo test. **(A)** AUCs for the diagnosis of advanced neoplasia. **(B)** AUCs for the diagnosis of CRC.

Regarding the four biomarker simultaneous semi-qualitative combo test, again hHb was the fecal biomarker with the best diagnostic accuracy with an AUC of 0.7 for advanced neoplasia and 0.783 for CRC, with no significant differences found with the quantitative FIT test. Even combining the four biomarkers, the AUCs were not significantly higher than the AUCs of hHb or quantitative FIT alone. The AUC of FIT compared with the combo test for both advanced neoplasia and CRC diagnoses are represented in Figure 3.

Analyzing each biomarker individually, hTf showed an acceptable diagnostic accuracy, both for advanced neoplasia (AUC 0.625) and CRC detection (AUC 0.764). As mentioned before, the AUC of hTf for diagnosis of advanced neoplasia was significantly lower than the AUC of hHb, although this difference was not statistically significant for CRC diagnosis. This may be due to the low number of CRC cases in our cohort (*n* = 22). Similar results were reported by Gies et al. although using a quantitative fecal transferrin test (25). Testing hCp and hLf showed no differences in positivity rates between advanced neoplasia and non-advanced neoplasia patients. Furthermore, both tests had an AUC slightly higher than 0.5. Therefore, they cannot be considered as useful tests in this setting (30). These findings are consistent with previous studies (24, 27). However, it is important to note that using either hHb alone or combined with hTf, 2 CRC would have been underdiagnosed and only the combination of hHb + hCp (likewise quantitative tests) detected all CRC cases (NPV 100%).

When comparing the quantitative tests with the semi-qualitative tests, the major difference was detected between quantitative FC and the semi-qualitative hCp test, especially for CRC diagnosis, as FC underdiagnosed 8 CRC cases (sensitivity 63.63%), whereas the hCp semi-qualitative test only missed one case (sensitivity 95.45%). Differences in FC levels are commonly found between assays from different manufacturers, increasing the difficulty of obtaining high quality evidence when comparing results of different FC tests (26). Nevertheless, neither of the two calprotectin tests showed an adequate diagnostic accuracy in this setting.

The main limitation of this study is that we have studied a selected population who had previously undergone a FIT and it was positive for a cut-off point of 20 μg/gr. Therefore, the results cannot be truly extrapolated to an average-risk screening population. This design was chosen so that the study could be conducted in parallel with the CRC screening program in our area, in which all the average-risk population undergoing colonoscopy have a previous positive FIT. It is noteworthy to mention, as another limitation, that the FIT used in our study is a different test that the used in the CRC screening program, although both are quantitative immunochemical tests. Further research repeating the same FIT may be necessary to confirm these findings.

Nevertheless, the main strength of the study is that it provides relevant information about the diagnostic accuracy of different fecal biomarkers, for which there is scarce evidence available at the moment, comparing its performance in the same population. Few studies analyzing different biomarkers individually in average risk population have been published so far. We have not found any study comparing the diagnostic accuracy of these four tests and their combinations. Thus, this study provides new data, highlighting the superiority of FIT compared with other biomarkers in this setting.

As a conclusion, in a selected average-risk population with a previous FIT test, fecal occult blood tests appear to be the best fecal biomarkers for the diagnosis of CRC and advanced adenoma. None of the other biomarkers explored, or their possible combinations, showed a better diagnostic accuracy than fecal hemoglobin, detected either with a quantitative FIT test or with a semi-qualitative test. Only fecal transferrin showed an acceptable diagnostic accuracy, a finding that can lead to further investigation in this setting.

## Data Availability Statement

The raw data supporting the conclusions of this article will be made available by the authors, without undue reservation.

## Ethics Statement

The studies involving human participants were reviewed and approved by CEICA–Regional Ethical Committee of Aragón. The patients/participants provided their written informed consent to participate in this study.

## Author Contributions

ÁL, FG, CS, and AL designed the study, interpreted data, and drafted the manuscript. GH-M collected and analyzed data, and drafted the manuscript. RV, NS, MN, CA, and ML collected data. EC and SA performed the biochemical analysis. All authors have revised the manuscript and have contributed to its intellectual content.

## Conflict of Interest

The authors declare that the research was conducted in the absence of any commercial or financial relationships that could be construed as a potential conflict of interest.
